# Scaling up proven public health interventions through a locally owned and sustained leadership development programme in rural Upper Egypt

**DOI:** 10.1186/1478-4491-8-1

**Published:** 2010-01-19

**Authors:** Morsi Mansour, Joan Bragar Mansour, Abdo Hasan El Swesy

**Affiliations:** 1 Management Sciences for Health, Cambridge, Massachusetts, USA; 2 Aswan Health and Population Directorate, Ministry of Health and Population, Aswan, Egypt

## Abstract

**Introduction:**

In 2002, the Egypt Ministry of Health and Population faced the challenge of improving access to and quality of services in rural Upper Egypt in the face of low morale among health workers and managers.

From 1992 to 2000, the Ministry, with donor support, had succeeded in reducing the nationwide maternal mortality rate by 52%. Nevertheless, a gap remained between urban and rural areas.

**Case description:**

In 2002, the Ministry, with funding from the United States Agency for International Development and assistance from Management Sciences for Health, introduced a Leadership Development Programme (LDP) in Aswan Governorate. The programme aimed to improve health services in three districts by increasing managers' ability to create high performing teams and lead them to achieve results.

The programme introduced leadership and management practices and a methodology for identifying and addressing service delivery challenges. Ten teams of health workers participated.

**Discussion and evaluation:**

In 2003, after participation in the LDP, the districts of Aswan, Daraw and Kom Ombo increased the number of new family planning visits by 36%, 68% and 20%, respectively. The number of prenatal and postpartum visits also rose.

After the United States funding ended, local doctors and nurses scaled up the programme to 184 health care facilities (training more than 1000 health workers). From 2005 to 2007, the Leadership Development Programme participants in Aswan Governorate focused on reducing the maternal mortality rate as their annual goal. They reduced it from 85.0 per 100,000 live births to 35.5 per 100,000. The reduction in maternal mortality rate was much greater than in similar governorates in Egypt. Managers and teams across Aswan demonstrated their ability to scale up effective public health interventions though their increased commitment and ownership of service challenges.

**Conclusions:**

When teams learn and apply empowering leadership and management practices, they can transform the way they work together and develop their own solutions to complex public health challenges. Committed health teams can use local resources to scale up effective public health interventions.

## Introduction

For ministries of health around the globe, well led and managed health programmes are crucial for producing health improvements that can be maintained. In Aswan Governorate in Egypt, a locally led and sustained leadership development programme (LDP) has empowered health workers in more than 180 primary health care facilities to contribute to improvements in health. The participants in this programme did what many others have been unable to do: when external support ended, they took a successful programme and expanded it.

With the benefit of many years of donor funding, Egypt succeeded in reducing the maternal mortality ratio (MMR) from 174 per 100,000 live births in 1992-1993 to 84 in 2000. The rapid decline in the MMR was due to factors that included "improved access to and quality of maternal and reproductive health services, reduced fertility rates, prenatal care utilization and skilled attendance at delivery" [1].

In spite of many improvements, however, gaps remained in access to and quality of services, especially in underdeveloped governorates. In Upper Egypt the MMR averaged 89 per 100,000 in 2000, while urban Egypt had an MMR of 46 [1]. Aswan, a governorate of more than two million people, is typical of the governorates in Upper Egypt, with a rural and mostly impoverished population.

Weaknesses in management at the local level are a major barrier to improved health outcomes. In 2007 the World Health Organization (WHO) noted that: "Weaknesses in general managerial capacity at all levels of the health system, but especially at the local level, are widely cited as a binding constraint to scaling up health services and achieving the MDGs. Scaling up depends on having some key resources, but it also depends to a large degree on how those resources are managed" [2].

Although management strengthening is needed, "attempts to strengthen district level health teams have often been limited by the policy and practice of national level government and donor agencies ... [and] by the extent to which donor-supported programmes were still based on standardized models which did not allow for varying and complex environments at district level" [3].

In 2002 the Population Sector of the Egyptian MOHP identified "the lack of commitment of front line health workers" as one of its greatest challenges. After Dr. Morsi Mansour, the Coordinator of Population and Family Planning Projects in Upper Egypt for the MOHP attended the Implementing Best Practices Conference and heard about the leadership and management practices presented by Management Sciences for Health (MSH), the MOHP asked the United States Agency for International Development (USAID) Office of Population to fund a one-year LDP in Aswan.

## Case description

The LDP differed from previous leadership training programmes, which had targeted only senior-level leaders. Its methodology is based on teams of health workers working together on service delivery challenges in their workplaces, with support and feedback from local managers.

**Figure 1:**
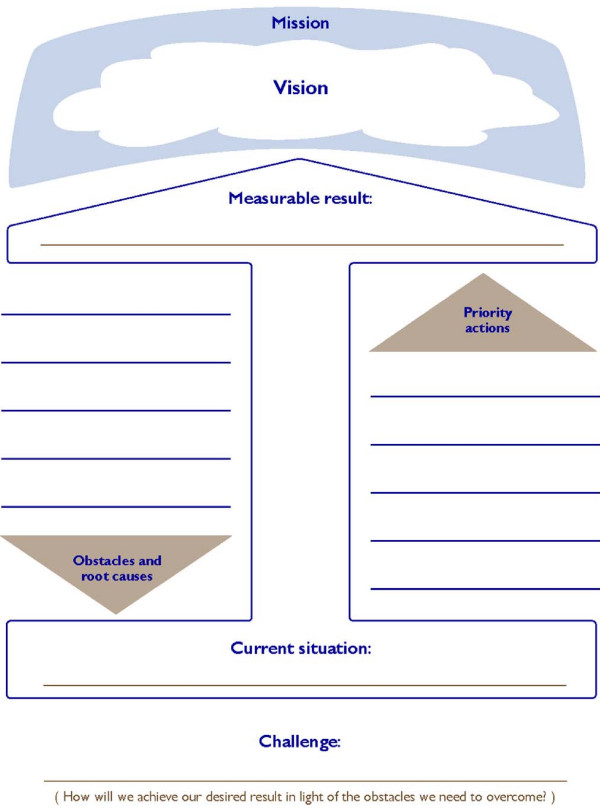
**The challenge model**.

In June 2002, with support from Dr Ayman Ragab, General Director of Health for the governorate of Aswan, the LDP pilot, co-led by MSH and the MOHP, was launched for 10 teams of doctors, nurses and midwives. The teams came from five primary health units, three districts and one rural hospital and included one team of governorate managers. Over several months, these teams participated in four one- or two-day workshops, learning leadership and management practices (see Figure 1) and applying simple planning and performance improvement tools to identify and address their challenges.

The purpose of the LDP was to empower local managers and health workers to improve the quality and accessibility of health services. The programme supported district and health facility teams in leading performance improvement projects to address service delivery challenges, increasing their skills in mobilizing local resources, monitoring results and improving the climate in their work groups and workplaces.

**Figure 2:**
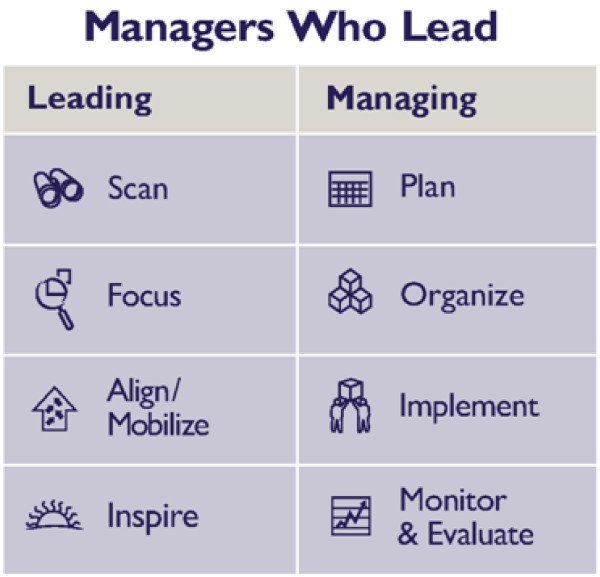
**Leading and Managing Practices Framework**.

The LDP used two tools to guide the work. Teams used the Challenge Model to work together towards a shared vision of the health outcomes they desired. The second tool, the Leading and Managing Practices Framework, provided a simple way to understand the leadership and management activities required to enable others to face challenges and achieve results (Fig 2).

Each team assembled baseline data and established a measurable performance goal. The teams designed projects to increase access to family planning services and prenatal and postpartum visits and carried out the projects in their clinics or districts. MOHP managers led monthly meetings to support the teams. Team meetings led by participants involved clinic staff--not just the LDP participants--in designing and carrying out the performance improvement projects.

Participants learned essential leading and managing practices: how to scan their environment and focus on a priority challenge; align and mobilize their teams and their stakeholders; and inspire each other. They learned to manage their resources--to plan, organize and implement activities--and to monitor and evaluate their results (see Fig 2).

The challenges required them to work together as aligned teams for the first time:

"Before the programme, if I needed something I would put in a request and ... if no one responded to my request, I would do nothing. ... Now our thinking is different. We try to do everything we can to help our patients and clients, not just our assigned tasks," said Jasmine Boshra Abdollah, a nurse in the Maternal and Child Health Center in Kom Ombo.

To build sustainability into the LDP workshops and team meetings, they were led in Arabic by MOHP facilitators. Exercises were reviewed by Ministry facilitators to ensure that they would be understood and accepted in the local context. Local managers facilitated short sessions of the programme and led district meetings between workshops to reinforce learning.

## Discussion and evaluation

At the end of the first year, eight of the 10 health teams had achieved 95% or more of their desired results and had selected a new challenge without prompting [4]. Three districts--Aswan, Daraw and Kom Ombo--increased the number of new family planning visits by 36%, 68% and 20%, respectively, compared to the same period the year before. Three teams achieved notable increases in the average number of prenatal care visits per client. Gaafra Health Centre achieved an average of 3.6 postpartum visits per client as of the end of June 2003, up from 0.2 visits in June 2002 [4].

MSH conducted studies in 2003, 2004 and 2005 to evaluate the results and learn more about the expansion of the self-directed programme. In 2003, at the end of the one-year pilot, they found that:

• All teams had changed from complaining about problems to identifying actionable challenges they could address.

• All teams collected complete or partial data on their selected challenges and used the data to prepare written action plans with measurable outputs, time frames and defined human and financial resources. There had been a major change, from merely sending routine reports to the next level to analysing the data to monitor progress and understand their challenges.

• All action plans used existing resources available to the teams; none required new human or financial resources.

Teams from the programme were eager to go to Cairo to present their success to the Ministry and demonstrate that by improving their own leadership they had improved service indicators. Nurses were among the proudest presenters.

### Sustaining and scaling up the programme

When USAID funding ended after one year, in 2003, Dr Abdo El Swesy, an obstetrician/gynaecologist from Kom Ombo District Hospital, and Dr Mohamed Souror, the District Family Planning Director from Kom Ombo District, convened a group of participants to discuss how to continue without outside funding. They faced many challenges initially. In the donor-funded LDP pilot, participants had attended meetings in hotels and received allowances for food and transportation. In the second year, meetings were held at health facilities, and the costs of materials and transportation came out of facilitators' and participants' pockets. Nevertheless, participation remained high. Using a 10-page booklet of handouts and modules adapted from the original programme, facilitators from the three districts took the revised programme to 15 new health facilities.

In the second year, teams from Aswan District raised the number of prenatal visits per woman from 1.3 to 3.7, and child care visits increased from 1.1 to 3.5. These improvements helped to change clients' perceptions of the health facilities and workers and influenced other health practices positively. The second generation of the Kom Ombo team created a new medical information system, and teams continued to increase the use of contraceptives, including condoms, pills, injectables and IUDs [4]. To achieve this, they implemented low-cost activities that could be accomplished by clinics using their own existing resources, including home visits, training for service providers and health education for women, men, and community leaders.

In the third year, the LDP scaled up to 100 health facilities and by 2005 it had reached all 184 primary health facilities--including more than 1000 health workers--in the governorate. Thirty-five local facilitators were developed over five 'generations'.

In 2004, an evaluation found that: "The LDP is perceived as a powerful tool to improve performance by all participants. The programme's participatory approach has enabled front line service providers to actively participate in discussions and ... in the design and implementation of their own small-scale service delivery improvement projects, as opposed to conducting projects 'imposed' by higher levels of the health system. This has contributed substantially to participants' enthusiasm and ownership of their service delivery challenges" [5].

### The LDP's role in decentralization

In 2005 an evaluation report found that: "Service providers who participated in this programme improved their commitment and love of their jobs...They act as partners to implement plans in their clinics rather than implementing plans put in place by somebody else. Trained health teams at the clinic level solve their own problems and don't wait for central level management to solve their problems" [6].

The evaluation also found that everyone who shared in the programme's implementation was motivated, the programme was easy to implement and it was adjustable, meaning that it was possible to add to it or to take things out as necessary [6].

The facilitators had problems that included:

• advocating the programme to higher management at the governorate or central level;

• finding time to expand the programme when the core team of facilitators became very busy (in new management positions);

• addressing the turnover of doctors during the training. When this happened health workers in the facilities had to bring new doctors up to date to be able to work with the rest of the team.

### Focusing on a governorate-wide challenge: reducing the maternal mortality rate

After training all health facilities in the governorate, in 2005 the Aswan LDP facilitators chose the governorate-wide challenge of reducing the MMR from 85 per 100 000 live births to 50. To accomplish this, LDP facilitators brought Safe Motherhood Committees to every district in the governorate. (These committees expanded on governorate-level committees initiated by the USAID Healthy Mother/Healthy Child Project.) District committees scanned to discover the factors contributing to maternal deaths in their areas. They focused on two factors that were within their control: (1) immediate transport of haemorrhaging women to a district hospital, and (2) a requirement that hospital physicians diagnose and treat in a team of three to prevent unnecessary haste and foster good decision-making under pressure. This requirement reduced unnecessary procedures and complications. [7]

Focusing on these two factors contributed to reducing the MMR for the entire governorate--a population of over two million. From 2006 to 2007, the Aswan Governorate reduced the MMR further, from 50.0 per 100,000 live births to 35.5. This figure continues to drop at a rate faster than in comparable governorates in Egypt. Qena is a governorate in Upper Egypt with similar economic and social conditions and similar amounts of MOHP and donor-funded programmes. In 2006 the MMR in Qena was 52.7 per 100,000, and in 2007 it was 52.0 per 100,000.

### Developing a new generation of leaders for the health system

Since completing the programme, more than 20 LDP facilitators and participants have taken leadership roles in the Aswan health directorate. The commitment of the health workers in Aswan and the results they have achieved over six years have come to the attention of the central MOHP, and other governorates across Egypt are requesting the LDP for their health teams.

### Scaling up to new areas and countries

Aswan facilitators have transferred LDP approaches to other governorates in Egypt. Through TAHSEEN, a USAID-funded project implemented in Egypt by the Catalyst Consortium, the Upper Egypt governorates of Minya, Bani Swaif and Fayoum were trained in LDP approaches and tools.

In 2005, Ministry of Public Health officials from Afghanistan went to Aswan to learn the LDP approach. They replicated the programme in five Afghan provinces that year. Now the LDP is used to improve service results in over 100 health facilities in 13 provinces across Afghanistan.

The LDP has been transferred to 35 developing countries around the world, and is used to scale up a variety of proven public health interventions. LDP tools and approaches are also being introduced into medical and public health curricula in Africa, Latin America and the Eastern Mediterranean.

## Conclusions

The LDP has scaled up with local resources because it uses a simple process of working with teams over time to focus on real health results, developing leaders at all levels of the health system and enabling local health managers to own the development process. It provides a pathway for teams of health managers and providers at all levels to lead and manage to improve performance.

Local leaders in Egypt saw that the programme produced results through this process and that it was easily transferred with local ministry staff as facilitators. They found inspiration and support in situations that were previously filled with despair. When technical assistance is seen as a resource, but not the source, of improved health, local leaders are free to be that source--to choose, use, 'own' and sustain necessary improvements in health care for their communities.

### Lessons learnt

To scale up a sustainable process for service improvement using available resources, we recommend the following.

• Empower front-line managers and their teams with leading and managing practices and a simple process for improving service results that is based on their shared vision of the health outcomes they want to achieve.

• Ensure that programme materials, processes and tools are simple and easy to adapt and contribute to measurable results that people care about.

• Support managers and their teams in playing the lead role in facilitating, adapting and sustaining health improvement programmes.

## Competing interests

MM and JBM are employed by Management Sciences for Health (MSH). MSH financed the writing of this article and the article processing fee.

## Authors' contributions

MM organized the technical and other resources to lead the original LDP in Egypt. JBM brought the applied transformational leadership models and practices to Egypt and worked with MM and a team of Egyptian consultants to design the contents of the LDP. AES led the scale-up process of the LDP in Aswan- including the adaptation of local training materials and development of 35 local facilitators. All authors contributed to and approved the final manuscript.

## Authors' information

From 2000-2003 MM was Coordinator of Family Planning and Reproductive Health in Upper Egypt, Ministry of Health and Population, Egypt; he is currently a Leadership Development Specialist at Management Sciences for Health, Cambridge, Massachusetts, United States of America. JBM is a Leadership Development Consultant for Management Sciences for Health. In addition to his post with the Ministry of Health and Population, Egypt, AES is also Director of the Infection Control Department and obstetrician and gynaecologist, Kom Ombo District Hospital, Aswan, Egypt.
